# Hypoalbuminemia Is a Hepatocellular Carcinoma Independent Risk Factor for Tumor Progression in Low-Risk Bridge to Transplant Candidates

**DOI:** 10.3390/cancers14071684

**Published:** 2022-03-25

**Authors:** Kelley G. Núñez, Tyler Sandow, Jai Patel, Mina Hibino, Daniel Fort, Ari J. Cohen, Paul Thevenot

**Affiliations:** 1 Institute of Translational Research, Ochsner Clinic Foundation, New Orleans, LA 70121, USA; kelley.nunez@ochsner.org (K.G.N.); jai.patel@ochsner.org (J.P.); mina.hibino@ochsner.org (M.H.); acohen@ochsner.org (A.J.C.); 2 Department of Radiology, Ochsner Health, New Orleans, LA 70121, USA; tyler.sandow@ochsner.org; 3 Center for Outcomes Research, Ochsner Clinic Foundation, New Orleans, LA 70121, USA; daniel.fort@ochsner.org; 4 Multi-Organ Transplant Institute, Ochsner Health, New Orleans, LA 70121, USA; 5 Faculty of Medicine, The University of Queensland, New Orleans, LA 70121, USA

**Keywords:** HCC, liver-directed therapy, albumin, liver transplantation

## Abstract

**Simple Summary:**

With the increasing rates of early-stage hepatocellular carcinoma (HCC) diagnosis, patients are being diagnosed with less tumor burden and low biomarker levels. Assessing HCC prognosis in these patients is challenging as most prognosis strategies are based on elevated tumor burden and biomarkers. In this study we focus on albumin levels prior to HCC treatment based on the established roles of albumin in HCC incidence and overall survival outcomes. The role of albumin as a risk factor for HCC progression was investigated with the goal of demonstrating that albumin could identify optimal candidates for treatment in early-stage HCC. These results may also impact the field by identifying patients with advancing cirrhotic disease who require more aggressive approaches to HCC treatment.

**Abstract:**

Due to active hepatocellular carcinoma (HCC) surveillance, many patients are diagnosed with early-stage disease and are usually amendable to curative treatments. These patients lack poor prognostic factors associated with Milan Criteria and alpha fetoprotein (AFP) biomarker levels. There are currently limited strategies to assess prognosis in the patients who remain at risk of post-treatment HCC progression. In a cohort of liver transplant (LT) candidates with HCC, this study seeks to identify factors prior to liver-directed therapy (LDT) associated with time to progression (TTP). This is a retrospective analysis of prospectively collected data from LT candidates with recently diagnosed HCC and receiving LDT as a bridge to LT at three interventional oncology programs within a single system (*n* = 373). Demographics, clinical hepatology and serology, and factors related to HCC burden were extracted and analyzed for associations with TTP risk. Albumin level below the cohort median (3.4 g/dL) emerged as an independent risk factor for TTP controlling for AFP > 20 ng/mL as well as Milan, T-stage, and Barcelona Clinic Liver Cancer (BCLC) stage individually. In modality-specific subgroup survival analysis, albumin-based TTP stratification was restricted to patients receiving first cycle microwave ablation (*p* = 0.007). In *n* = 162 patients matching all low-risk criteria for Milan, T-stage, BCLC stage, and AFP, the effect of albumin < 3.4 g/dL remained significant for TTP (*p* = 0.004) with 2-year TTP rates of 68% (<3.4 g/dL) compared to 95% (≥3.4 g/dL). In optimal bridge to LT candidates with small HCC and low AFP biomarker levels, albumin level at treatment baseline provides an HCC-independent positive prognostic factor for risk of HCC progression prior to LT.

## 1. Introduction

Hepatocellular carcinoma (HCC) is a leading cause of cancer-related mortality [1]. Cirrhosis is the predominant risk factor for developing HCC and although many patients remain well compensated, extensive cirrhosis often precludes surgical resection. HCC is generally diagnosed within Milan Criteria for patients with end-stage liver disease and thus they are eligible for liver transplantation (LT). While awaiting LT, it is critical to maintain the HCC burden within Milan by using liver-directed therapy (LDT) [2]. Retrospective studies have shown that combinations of the Model of End Stage Liver Disease (MELD)c score, Milan Criteria, and alpha fetoprotein (AFP) biomarker levels at diagnosis can be used to stratify an ideal bridge to LT candidates [3]. As HCC surveillance and early-stage diagnosis continue to improve [4], modern HCC cohorts routinely report baseline HCC burden > 75% within Milan, median AFP values < 20 ng/mL, and MELD-Na scores < 15. Consequently, early-stage diagnosis has created a strong downward pressure on established risk thresholds, which has complicated assessing baseline bridge to LT prognosis in patients with end-stage liver disease not suitable for surgical resection.

Beyond baseline factors, an objective response rate [5] to first cycle LDT remains the most powerful prognostic indicator of bridge to LT outcomes [6]. Unfortunately, 25–40% of patients do not achieve an objective response to first line LDT and may experience rapid intra- or extra-hepatic spread resulting in progression beyond LT criteria [7]. In optimal patients lacking Milan- or AFP-based risk factors, there are limited options to assess HCC biological aggression, LDT prognosis, and risk of post-LDT progression.

With the emergence of checkpoint inhibitor immunotherapy, the immune landscape as a barrier to cancer therapy has come into focus. The immunological landscape in HCC is largely driven by the progression of cirrhosis through a process called cirrhosis-associated immune dysfunction (CAID). The spectrum of CAID extends from an early proinflammatory phase in compensated cirrhosis to an immunosuppressed phase in decompensated cirrhosis [8]. Although the role of HCC in this paradigm remains unclear, an immunocompromised state would likely favor HCC development and limit the ability to generate an effective anti-tumoral immune response following LDT. CAID progression is marked by declining serum albumin levels and likely indicates a more advanced disease than suggested by the patient’s MELD score. Chronic inflammation and tumor progression can increase albumin degradation and catabolism, both decreasing the functional capability of albumin as well as causing an overall decrease in total circulating albumin (reviewed in [9,10]). As endotoxin levels increase due to portal hypertension, the capacity for albumin to bind endotoxin is reduced, leading to increased prostaglandin E2 bioavailability and creates a state of immune paralysis [11]. These immune-linked functions of albumin may play a key role in HCC behavior following LDT, including the risk of HCC progression following LDT.

In this single center retrospective analysis, we focus on recently diagnosed HCC patients receiving LDT as a bridge to LT and examine the role of albumin in HCC progression risk. The role of albumin as an independent factor for time to progression was investigated across LDT treatment modalities as well as in optimal bridge to LT candidates with small HCC burden and low AFP biomarker levels.

## 2. Materials and Methods

### 2.1. Study Design and Population

The study was designed as a retrospective analysis and approved by the Ochsner Clinic Foundation Institutional Review Board (protocol 2019.308, principal investigator Tyler Sandow) and in accordance with the ethical guidelines set forth by the Declaration of Helsinki. The cohort was assembled longitudinally by identifying patient encounters for LDT within the Ochsner Health System by surveying the electronic medical record database using EPIC Slicer Dicer software. Extracted records were then screened to yield patients with a recent HCC diagnosis confirmed by biopsy or triple-phase imaging in accordance with LI-RADS criteria who were also (i.) being evaluated with intention to treat by LT and (ii.) had been reviewed by the multidisciplinary HCC board and designated to receive LDT. The study criteria for intention to transplant were non-resectable HCC with extensive cirrhosis, Child Pugh A-B, T-stage T1-T2, Barcelona Clinic Liver Cancer (BCLC) A-B, and Eastern Cooperative Oncology Group (ECOG) 0–1 as assessed immediately prior to the first cycle of LDT.

### 2.2. Primary Outcome

The cohort was followed longitudinally to a primary endpoint of HCC progression beyond transplant criteria as determined by the multidisciplinary HCC board based on follow-up or surveillance triple-phase imaging. HCC progression was defined as being no longer a transplant candidate due to progression beyond Milan Criteria or failure to downstage within Milan Criteria. HCC progression was categorized as (i.) local HCC progression (including extrahepatic metastasis without new hepatic foci) or (ii.) new hepatic foci. The primary assessment of outcome was performed using time to progression (TTP) defined as the time from first cycle LDT until HCC progression. TTP analysis was censored for the following conditions: LT, election to pursue systemic therapy without tumor progression, lost to follow-up, all-cause mortality, or no evidence of HCC progression at the time of data analysis. Censoring date was defined at the time of the disqualifying event with exception to no evidence of HCC progression in which the censoring date was defined as the primary analysis date (1 December 2021).

### 2.3. Study Variables

General demographics, cirrhosis history, liver serology, and variables related to HCC diagnosis were extracted from the electronic medical record. Serology values were obtained from day-of-LDT labs. HCC size and overall burden were obtained from the initial multidisciplinary HCC board report prior to LDT. Indices for cirrhosis and HCC (Child Pugh, Milan, ECOG, T-stage, BCLC, and albumin-bilirubin (ALBI) grade) were calculated as defined in the literature. History of decompensation was defined as an episode of ascites, esophageal varices, or hepatic encephalopathy necessitating pharmacological (diuretics, lactulose, beta blockers) or surgical intervention (TIPS, banding, paracentesis) and occurring prior to the first cycle of LDT.

### 2.4. Liver-Directed Therapy Sites and Treatment Protocols

The interventional oncology sites were all located in Louisiana (Baton Rouge, New Orleans, Shreveport) and linked under a single liver transplant center (Ochsner Health Multi-Organ Transplant Institute, New Orleans, LA, USA). The multidisciplinary HCC board encompassed all interventional oncology sites and consisted of transplant hepatologists, transplant surgeons, interventional radiologists, and oncologists. Institutional criteria for LDT were (a) BCLC A-B, (b) Child-Pugh A-B, (c) non-resectable HCC, (d) without main portal vein thrombus or extrahepatic metastasis, (e) total bilirubin < 4 mg/dL, (f) serum creatinine concentration < 1.5 mg/dL, and (g) absent gross ascites by ultrasound or CT. Treatment approach and modality were determined by the committee and based upon patient-specific variables including performance status as well as size and location of both index and any secondary HCC. First cycle LDT included drug-eluting embolic transarterial chemoembolization (DEE-TACE), 90Yttrium transarterial radioembolization (90Y), or percutaneous microwave ablation (MWA). The study period overlapped a change in the institutional algorithm for first cycle treatment of HCC. Patients receiving first cycle LDT in 2016 received DEE-TACE. From 2017 to study conclusion, the institutional algorithm was MWA for an ablatable index HCC < 3 cm with 90Y as first cycle for non-ablatable index HCC. Patients with contraindication for both MWA and 90Y received DEE-TACE as first-line LDT. At the time of data analysis (1 December 2021), institutional experience for first cycle HCC treatment as a bridge to LT by modality was >10 years (DEE-TACE), >5 years (90Y), and >5 years (MWA).

DEE-TACE was performed using 100–300 μm drug-eluting beads (LC Bead, BTC, Lakeview, Riverside way, England) loaded with 50 mg doxorubicin per vial with the entire vial defined as the planned index treatment dose. All vessels feeding the areas of tumor were treated prior to follow-up imaging. 90Y was performed as a two-phase treatment, including a mapping angiogram with a calculation of lung shunt fraction followed by 90Y glass microsphere infusion (TheraSphere; Boston Scientific, Marlborough, MA, USA) [12]. Mapping angiography included vascular evaluation of the celiac artery, superior mesenteric artery, proper hepatic artery, and all feeding hepatic arteries to the areas of tumor. During treatment, all feeding vessels to areas of the tumor were treated with target radiation doses greater than 200 Gy. Ablation was performed using a high-powered, gas-cooled, multiple antenna-capable system (Neuwave Medical, Madison, WI, USA). Duration of treatment and power application were determined by the performing physician at the time of treatment and based on manufacturer guidelines with adjustments made for the tumor size or proximity to vulnerable structures. The ablative margin was set at >5 mm for all MWA procedures.

First cycle response to LDT was recorded as objective response rate [5] using the Response Evaluation Criteria in Solid Tumors modified for HCC (mRECIST) in all patients with treatment cycle follow-up imaging [13]. Follow-up imaging was modality-dependent and targeted to 30 days (DEE-TACE) or 60 days (90Y and MWA). In patients undergoing a predetermined treatment sequence with multiple cycles of LDT to the index lesion without intermittent follow-up imaging, the ORR was evaluated following the final treatment in the sequence. Treatment track after follow-up imaging was dictated by the multidisciplinary HCC board and mapped to the following treatment tracks: (a) satisfactory response to treatment or successfully down staged to within Milan Criteria with recurring 3 month surveillance until LT, (b) repeat treatment of the index HCC or non-index burden as a continued bridge to LT, (c) met censoring criteria for reasons not attributable to HCC progression beyond Milan Criteria, or (d) declared no longer a transplant candidate due to HCC progression or failure to downstage within Milan Criteria with referral for advanced stage treatment.

### 2.5. Statistical Methods

Data analysis was performed in JMP 13.0 (SAS Institute Inc.) with graphical output generated using Prism 8.0 (GraphPad Software Inc., San Diego, CA, USA). Continuous variables were expressed as median with interquartile (IQR) range. Categorical variables were expressed as number and percentage of the cohort unless otherwise noted. Univariate and multivariate Cox regression analysis was used to determine variables associated with TTP. Univariate continuous values reaching *p* < 0.050 were converted to quartiles and retested for significance prior to inclusion in the multivariate analysis. Component measures of HCC burden reaching significance in univariate analysis which are components of established clinical indices for HCC progression risk were excluded from multivariate analysis in favor of the clinical indices. Multiple multivariate models were utilized to account for overlapping factors within indices of Milan, T-stage, and BCLC. Albumin was investigated in multivariate models using both quartile and median split format to identify the optimal threshold for downstream analysis. Kaplan–Meier survival curves were generated in Prism and compared using log-rank tests. Differences in the component variables between albumin median splits were analyzed using Mann–Whitney, Fisher, and Chi-square tests.

## 3. Results

### 3.1. Cohort Demographics

**Figure 1:**
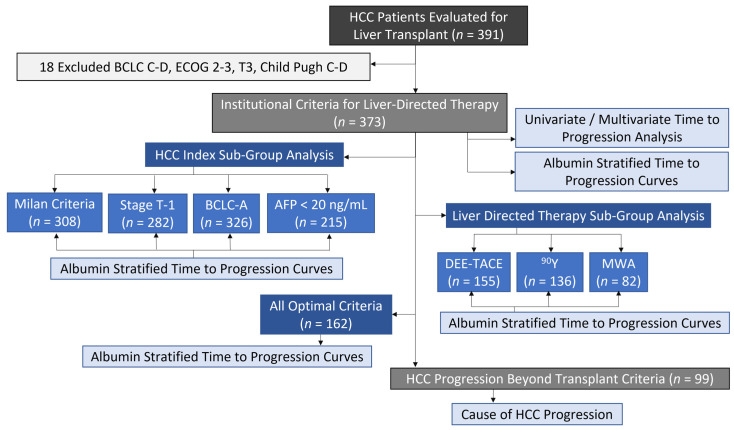
CONSORT Diagram of the Study Cohort. Outline of the study cohort exclusion criteria, analysis cohort characteristics, and subgroup analysis. Abbreviations: hepatocellular carcinoma (HCC), Barcelona Clinic Liver Cancer (BCLC), Eastern Cooperative Oncology Group (ECOG), alpha-fetoprotein (AFP), doxorubicin-eluting embolic transarterially chemoembolization (DEE-TACE), Yttrium-90 (90Y), microwave ablation (MWA).

Identified were 373 HCC patients matching institutional criteria for LDT and LT evaluation after excluding 18 candidates outside criteria (Figure 1). General demographics, baseline hepatology and HCC characteristics are summarized in Table 1. Chronic hepatitis C virus (HCV) was the predominant etiology of cirrhosis (208/373, 56%) with a median age of 63 years and mostly male (286/373, 77%). Overall, liver disease was well compensated with a median MELD-Na of 9 and Child Pugh A (284/373, 76%). The study focus on non-resectable early-stage HCC was reflected in the baseline tumor burden with a median index HCC size of 2.8 cm. The cohort was predominantly within Milan (308/373, 83%), stage T-1 (282/373, 76%), BCLC-A (326/373, 87%), and ECOG 0 (228/3773, 61%) with a median AFP of 13 ng/mL and 58% (215/373) having an AFP < 20 ng/mL.

### 3.2. Liver-Directed Therapy Baseline Factors Associated with Time to Progression

**Figure 2:**
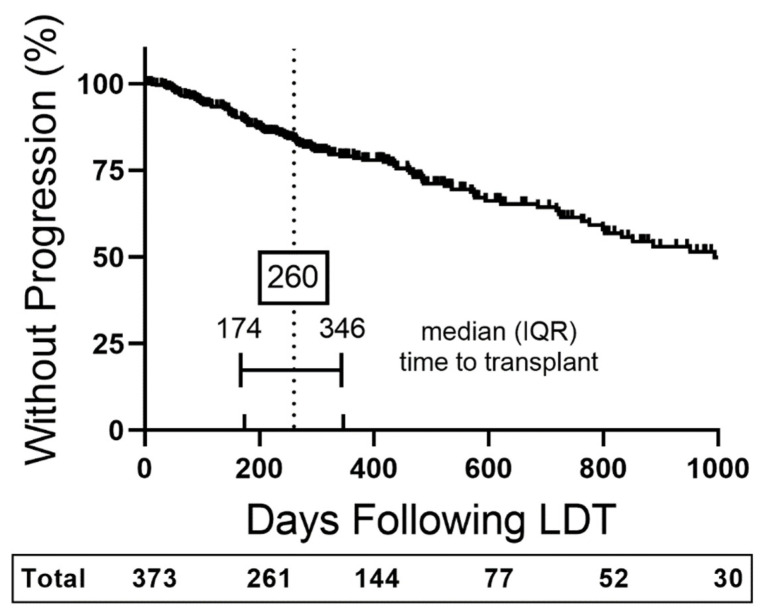
Study Cohort Overall Time to Progression Curve. Kaplan–Meier curve of the percentage of patients without hepatocellular carcinoma progression as a function of time after first cycle liver-directed therapy (LDT). Censored data is marked with a vertical upward dash. The total number of patients remaining in the analysis at each major x-axis time point is listed in the table below the plot. Interquartile range (IQR).

Median time to HCC progression was 994 days compared to a median time to LT of 260 days (Figure 2). The frequency of patients without HCC progression at the median time to LT was 84%. Continuous values of creatinine, albumin, and the INR were associated with TTP in univariate analysis; however, only albumin (*p* = 0.006) maintained an association after derivation to quartiles of ≤2.9, 3.0–≤3.4, 3.5–≤3.7, and >3.7 g/dL (Table 1). All HCC diagnostic factors and indices were associated with TTP with exception to the ECOG score. Multivariate analysis was performed using Milan, T-stage, and BCLC individually in combination with albumin quartiles and elevated AFP >20 ng/mL (Table 2). Albumin quartiles were associated with TTP risk regardless of how HCC burden was modeled. The risk associated with albumin was in decreasing from the 3.0 to ≤3.4 g/dL tier to the ≤2.9 g/dL, with hazard ratios [14] ranging from 2.1 (95% confidence interval, CI, 1.2–3.5) to 2.2 (CI 1.3–3.7).

Although bilirubin level was not associated with TTP (Table 1), we analyzed the distribution of ALBI grade among albumin quartiles (Table S1). Patients with albumin ≤ 2.9 g/dL were divided among ALBI Grade 2 (41/95, 43%) and Grade 3 (54/95, 57%). A median split of the cohort albumin level at <3.4 g/dL divided the population of ALBI Grade 2 patients, with 148/252 (58%) with albumin < 3.4 g/dL and 104/252 (42%) at ≥3.4 g/dL. To determine where a median split of albumin, containing much of the ALBI Grade 2 population, captured TTP risk in this population, we reproduced multivariate analysis of TTP analysis with albumin at a median split (Table 3) and those obtained using the ALBI Grade (Table 4). Modeling with a median split for albumin resulted in a small decrease in HR from 2.1–2.2 (quartiles) to 1.9–1.8 (median split) but remained statistically significant (*p* = 0.001 Milan Model, *p* = 0.005 T-stage and BCLC models). The ALBI Grade only remained in model for Milan and T-stage models, with *p* = 0.037 (HR 1.9 CI 1.4–6.3, Grade 2 to Grade 3 Milan Model) and *p* = 0.31 (HR 1.9 CI 1.3–6.0, Grade 2 to Grade 3 T-stage Model). Therefore, a median split of albumin was utilized for subsequent analysis.

### 3.3. Albumin-Based Stratification of Early-Stage HCC Outcomes

**Figure 3:**
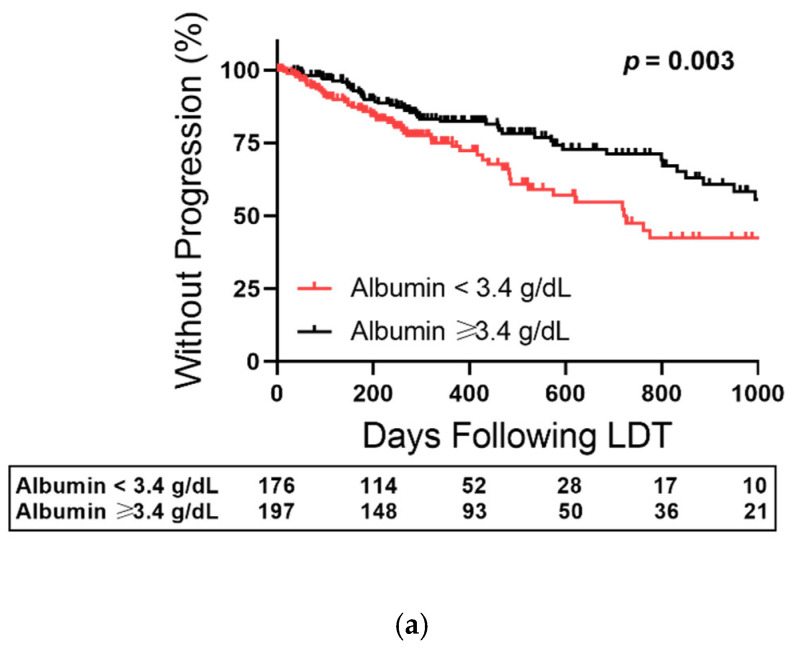

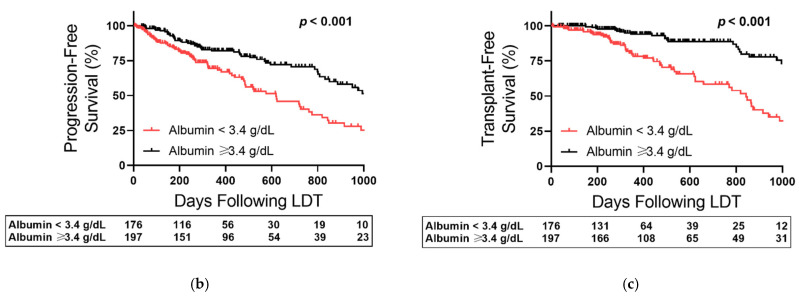
Albumin-Stratified Kaplan–Meier Curves of Bridge to Transplant Outcomes after First Cycle Liver-Directed Therapy (LDT). (**a**) Percentage of patients without hepatocellular carcinoma progression as a function of time and stratified at an albumin level of 3.4 g/dL. (**b**) Progression-free survival curves stratified by albumin level. (**c**) Transplant-free survival curves stratified by albumin level. Censored data is marked with a vertical upward dash. The total number of patients remaining in the analysis at each major x-axis time point is listed in the table below the plot. Significance was determined using the Log-Rank test.

The overall cohort was stratified based on 3.4 g/dL median split for albumin to test the role of albumin in time-dependent outcomes for early-stage HCC (Figure 3). Baseline albumin stratified TTP (*p* = 0.003), with 87% without progression with albumin ≥ 3.4 g/dL compared to 79% with albumin < 3.4 g/dL at the 260-day center median transplant time (Figure 3a). The albumin < 3.4 g/dL subgroup reached median TTP at 2 years after first cycle LDT (722 days) compared to 71% without progression having albumin ≥ 3.4 g/dL. Albumin similarly stratified progression-free survival with median survival of 1027 days for patients with albumin ≥ 3.4 g/dL and 619 days in those with <3.4 g/dL (Figure 3b, *p* < 0.001). Similarly, transplant-free survival was longer in patients with albumin ≥ 3.4 g/dL with a median survival of 1593 days compared to 843 days in those with <3.4 g/dL (Figure 3c, *p* < 0.001).

### 3.4. Albumin-Based Stratification of Outcomes in Low-Risk Early-Stage HCC

**Figure 4:**
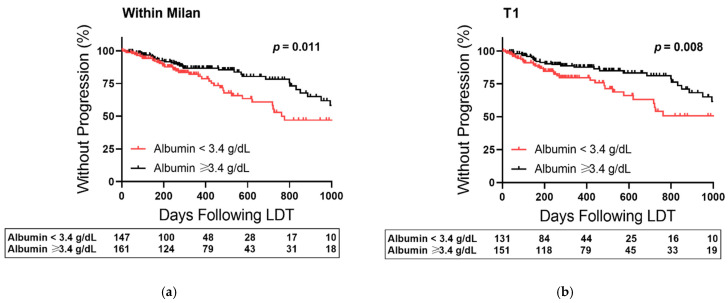

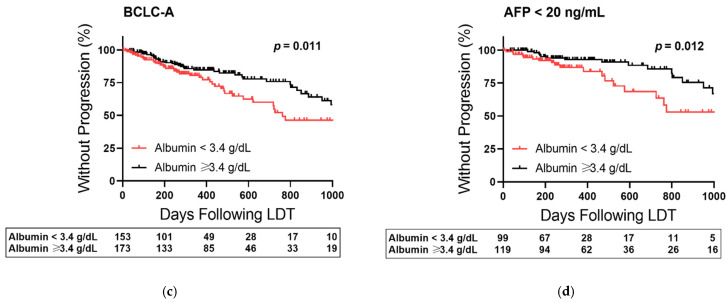
Albumin-Stratified Kaplan–Meier Curves of Time to Progression Based Upon HCC Burden and Biomarker Risk Factors following Liver-Directed Therapy (LDT). (**a**) Plot restricted to patients within Milan Criteria at diagnosis. (**b**) Plot restricted to patients with T1 stage burden at diagnosis. (**c**) Plot restricted to patients with Barcelona Clinic Liver Cancer (BCLC) Stage A disease at diagnosis. (**d**) Plot restricted to patients with a baseline alpha-fetoprotein (AFP) level < 20 ng/mL. Censored data is marked with a vertical upward dash. The total number of patients remaining in the analysis at each major x-axis time point is listed in the table below the plot. Significance was determined using the Log-Rank test.

The effect of baseline albumin stratification was then examined within the low-risk tier of each HCC index from the multivariate analysis (Figure 4). Albumin stratified TTP curves in patients within Milan (Figure 4a, *p* = 0.011), HCC burden stage T1 (Figure 4b, *p* = 0.008), BCLC-A (Figure 4c, *p* = 0.011), and with AFP < 20 ng/mL (Figure 4d, *p* = 0.012). In low-risk patients, the effect of albumin < 3.4 g/dL on HCC progression was most prominent approaching 20 months (620 days) after the first LDT cycle with 17% (within Milan), 20% (T1), 18% (BCLC-A), and 19% (AFP < 20 ng/mL) higher progression rates compared to albumin ≥ 3.4 g/dL.

### 3.5. Role of First Cycle Treatment Modality in Albumin-Based Stratification

**Figure 5:**
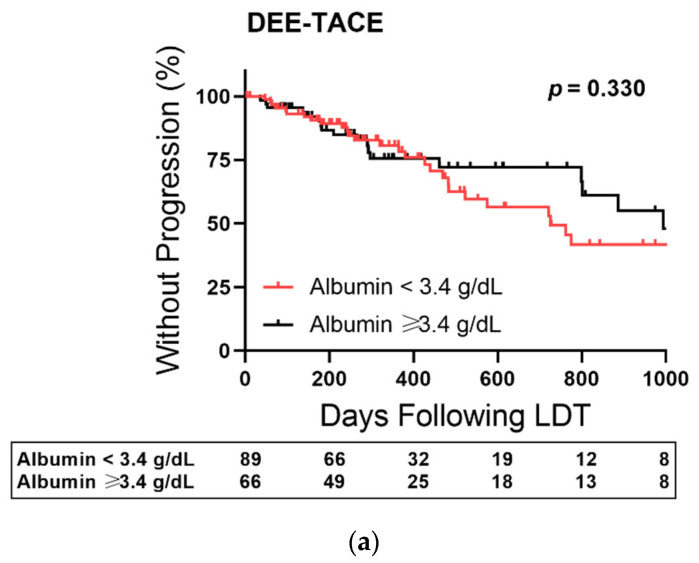

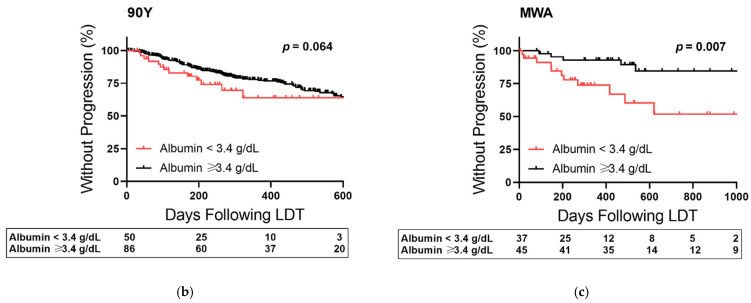
Albumin-Stratified Kaplan–Meier Curves of Time to Progression Based First Cycle Liver-Directed Therapy (LDT) Treatment Modality. (**a**) Plot restricted to patients treated with drug-eluting embolic transarterial chemoembolization (DEE-TACE). (**b**) Plot restricted to patients treated with Yttrium-90 (90Y). (**c**) Plot restricted to patients treated with microwave ablation (MWA). Censored data is marked with a vertical upward dash. The total number of patients remaining in the analysis at each major x-axis time point is listed in the table below the plot. Significance was determined using the Log-Rank test.

The cohort was then grouped based on first cycle LDT modality and TTP outcomes examined after stratifying for pretreatment albumin level (Figure 5). Log-rank testing of albumin stratification in the DEE-TACE (Figure 5a) and 90Y cohorts (Figure 5b) did not reach significance. However, albumin baseline was able to stratify TTP outcomes in patients receiving first cycle MWA (Figure 5c). Patients with albumin < 3.4 had a 15% higher progression rate at the 260-day median time to transplant and up to 30% at higher at 16 months following first cycle MWA.

**Figure 6:**
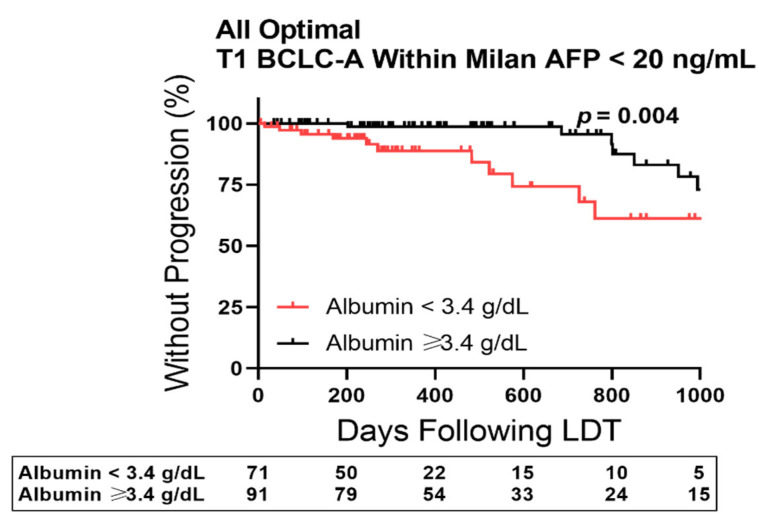
Albumin-Stratified Kaplan–Meier Curves of Time to Progression in Optimal Bridge to Liver transplant (LT) Patients. Plot restricted to patients with burden at diagnosis defined as T1 and within Milan Criteria, with Barcelona Clinic Liver Cancer (BCLC) Stage A disease, and having a baseline alpha-fetoprotein (AFP) < 20 ng/mL. Censored data is marked with a vertical upward dash. The total number of patients remaining in the analysis at each major x-axis time point is listed in the table below the plot. Significance was determined using the Log-Rank test.

MWA is institutionally restricted to patients with index HCC < 3 cm and patients in the cohort receiving MWA were more often unifocal (70/82, 85% compared to 212/290, 73%) with AFP < 20 ng/mL (53/82, 65% compared to 182/290, 56%). Therefore, a subgroup was constructed for patients matching each low-risk HCC criteria, resulting in a cohort of unifocal, small to intermediate size HCC with low AFP biomarker <20 ng/mL (Figure 6). Albumin stratification of TTP outcomes in these optimal patients revealed patients with albumin ≥ 3.4 g/dL prior to first cycle LDT had 1-year HCC progression rates < 2% compared to 11% with albumin < 3.4 g/dL.

### 3.6. Difference in Baseline Factors Prior to First Cycle LDT after Albumin Stratification

The clinical characteristics of the cohort at baseline were then reexamined after stratifying based on albumin level prior to first cycle LDT (Table 5). Median age was 2 years lower (*p* = 0.003), with a lower frequency of males (*p* = 0.002) in patients with albumin < 3.4 g/dL at baseline. Although there was no difference in cirrhosis etiology (*p* = 0.735), the rates of Child Pugh B were 43% higher (*p* < 0.001) and prior history of decompensation was 33% higher (*p* < 0.001) accompanied by a higher median MELD-Na score in patients with <3.4 g/dL albumin at baseline (*p* < 0.001). However, there were no significant differences in any measures of HCC burden or AFP biomarker levels. Based on LDT modality criteria, patients with low albumin more often received DEE-TACE first cycle (89/176, 51% compared to 66/197, 34%) and less frequently 90Y (50/176, 28% compared to 86/197, 44%). The modality-independent objective response rate was 10% higher in patients with albumin ≥ 3.4 g/dL (*p* = 0.058, excluding those with unavailable imaging).

## 4. Discussion

This study focuses on an early-stage HCC patient cohort undergoing LDT with intention to LT at a single health system with a unified treatment algorithm. The intention to treat approach provides a more comprehensive view of factors driving HCC progression while eliminating center-dependent factors related to time-to-treatment and waitlist strategies. To avoid excluding this intention to LT patients with treatment history prior to waitlist activation, first cycle LDT was set as time zero in the TTP analysis. Using baseline status at diagnosis, prior to first cycle LDT, we can focus on the growing population of early-stage HCC patients with initial burden within Milan Criteria and low levels of the AFP biomarker. Given the current clinical limitations in genetic and molecular characterization of biological aggressiveness in early-stage disease, this study focused on the role of albumin in HCC progression risk following first cycle LDT.

In a 5-year cohort of transplant candidates monitored from first cycle LDT, multivariate analysis of TTP revealed albumin level prior to first cycle was the only HCC-independent variable associated with TTP risk. Albumin is a well-known risk factors for developing HCC [14] and, when incorporated into the ALBI grade and several other prognostic scores, can stratify PFS and OS outcomes following LDT as well as OS and RFS after hepatectomy or LT (recently reviewed in [15,16,17,18]). There have been far fewer studies investigating a direct relationship between albumin and HCC progression independent of overall mortality.

Although the mechanistic process through which deteriorations in hepatic reserve drive tumor progression remain unclear, current evidence suggests these effects are mediated by CAID. CAID is characterized by immune exhaustion resulting in impaired innate and adaptive immunity through mechanisms linked to hypoalbuminemia [8,11,19]. Albumin was recently shown to play a tumor suppressor role in HCC by suppressing migration and invasion in HCC cell lines [20]. Albumin was also recently incorporated into the MELD 3.0 score [21] and our results suggest these changes may improve MELD-based prognosis of HCC progression compared to the MELD-Na. Beyond direct effects of albumin on HCC biology, the role of prostaglandin E2-mediated depletion of albumin stores in advancing CAID and its role in systemic immune suppression are becoming clearer [11,22,23].

Hypoalbuminemia (albumin < 3.4 g/dL) was more common in females with advancing cirrhotic disease (Child Pugh B, elevated MELD score, and history of decompensation) but not associated with etiology of cirrhosis, any direct or index radiographic measure of HCC burden, or AFP biomarker levels. Although the percentage of patients with HCV-cirrhosis was similar among albumin levels, a recent study showed lower albumin levels in patients with HCV viremia at HCC diagnosis compared to aviremic patients treated with direct-acting antivirals (DAA) [24]. In agreement with this report, 72% of HCV-HCC patients with albumin < 3.4 g/dL were viremic compared to 50% of HCV-HCC patients with albumin ≥ 3.4 g/dL. Other studies have shown DAA treatment leads to improved albumin levels [25,26] which may reduce post-LDT HCC progression risk and is the subject of further investigation.

ALBI grade has been validated for PFS and OS across the spectrum of HCC staging, but progression risk in multivariate analysis controlling for HCC burden and the AFP biomarker was restricted to the small percentage of ALBI Grade 3 patients compared to ALBI Grade 2. Additionally, bilirubin level was not associated with TTP risk in univariate or multivariate analysis. A median split for albumin was chosen for the study focus due to a higher significance and a lower 95% confidence interval compared to ALBI grade while also identifying TTP risk in both ALBI Grades 2 and 3.

In both the overall cohort and the optimal LT candidate subgroup, the effect of albumin was most prominent beyond center median time from first cycle LDT to LT (>260 days). The study center is characterized as a short wait time transplant center, with most HCC patients receiving LDT prior to waitlist activation. First cycle LDT to transplant time, therefore, provides a more accurate representation of the overall care timeline compared to time on the transplant waitlist. This data suggests that HCC progression risk increases specifically in patients with hypoalbuminemia as time to LT is extended beyond the median. Although the median MELD score was higher in patients with albumin < 3.4 g/dL, this did not translate into a decrease in time from first cycle LDT to LT, with albumin < 3.4 g/dL at 248 days compared to 274 days with albumin ≥ 3.4 g/dL.

The potentially confounding role of multiple treatment cycles leading to extended wait time and increased progression risk in optimal patients with albumin < 3.4 g/dL may be minimal as the first cycle ORR was similar across albumin levels. Further, there was no significant difference in the rates of index progression beyond transplant criteria versus new disease leading to progression beyond transplant criteria, suggesting new disease was not the primary contributing factor to HCC progression risk in patients with albumin < 3.4 g/dL. A potentially confounding role for duration of first cycle response leading to extended wait time is under further investigation. The data, however, suggests that albumin may play a critical role identifying progression risk in optimal LT candidates, where the 2-year progression rates were 32% in patients with albumin < 3.4 g/dL compared to 5% if albumin ≥ 3.4 g/dL.

To our knowledge, this is the first study to use hypoalbuminemia to stratify progression outcomes for first cycle MWA in early-stage HCC. The current clinical practice guidelines of the AASLD and EASL [2,27] recommend MWA for the treatment of HCC tumors ≤ 3 cm. As a result, first cycle MWA cohorts are biased to smaller tumor burden and may have more preserved liver function compared to patients receiving first cycle intra-arterial therapies. MWA treats the tumor by causing coagulative necrosis with limited damaged to surrounding non-tumor tissue [28], whereas DEE-TACE and 90Y trigger tumor death through programmed cell death pathways leading to apoptosis [5]. Although our understanding of antigenicity, the generation of neoantigens, and adjuvanticity, the capacity to promote effect antigen presentation, in the context of LDT remains limited; they may play a more critical role in HCC outcomes following MWA. If hypoalbuminemia in these patients is a manifestation of CAID-mediated immune paralysis, their ability to mount an effective post-LDT antitumoral immune response may be extremely limited and drive the risk of HCC progression following MWA.

The study cohort received LDT at multiple centers within a single health system in a region with a higher frequency of disadvantaged populations and more advanced liver disease at HCC diagnosis. The study period encompassed a changed in the center treatment algorithm, where 90Y replaced DEE-TACE as the preferred treatment approach to non-ablatable HCC > 3 cm. Although an increasing number of centers have adopted 90Y for bridge to LT, the experience of the interventional radiologists and the use of personalized dosimetry can significantly affect 90Y outcomes. There are biases in baseline liver function and HCC burden among patients grouped by first cycle LDT modality, and this bias was addressed by analyzing albumin-dependent progression outcomes within each treatment modality. Further, time to follow-up and time to re-treatment vary based on LDT modality and the role of LDT-specific treatment timeline and compliance is under further investigation. The retrospective study design limited the ability to confirm a direct association between CAID mediators and hypoalbuminemia, which can be addressed in a prospective study design. Although changes in albumin level post-LDT could be associated with progression risk, post-procedure decreases in albumin have been described in the LDT literature [29,30] and guided the study focus on pre-LDT albumin levels. Finally, the intention to LT study design allows for extended TTP outcomes due to patient preference to defer LT as well as HCC-independent changes in other comorbidities that may preclude LT. The findings and their implications should be discussed in the broadest context possible. Future research directions may also be highlighted.

## 5. Conclusions

In conclusion, this study finds that hypoalbuminemia (<3.4 g/dL) is an independent risk factor for tumor progression in early-stage, non-ablatable HCC. Hypoalbuminemia in these recently diagnosed patients is independent of HCC burden but highly associated with a history of decompensation of cirrhosis. TTP risk with hypoalbuminemia is most pronounced in patients with extended time to LT following first cycle LDT. The effect of hypoalbuminemia is most pronounced in patients receiving first cycle MWA and remains an indicator of progression risk in optimal bridge to LT candidates with well-preserved liver function, limited HCC burden, and low AFP levels. If a direct link between early-stage HCC with hypoalbuminemia and immune paralysis in CAID can be established, strategies could be developed to reverse and/or promote effective antitumoral immunity post-LDT and improve TTP outcomes.

## Figures and Tables

**Table 1:** Clinical characteristics and time to progression analysis in an intention to bridge to liver transplant cohort.

Parameters	Data	*p*-Value
Cohort, *n*	373	
Study period, date range	21 April 2016–4 November 2021	
General Demographics		
Age, median (IQR)	63 (60–67)	0.815
Legal sex, *n* male (%)	286 (77)	0.784
Hepatology at Diagnosis		
Cirrhosis etiology, *n* (%)		0.950
HCV	208 (56)	
NASH	52 (14)	
HCV + ALD	44 (12)	
ALD	34 (9)	
Other	35 (8)	
Child Pugh, *n* (%)		0.122
A	284 (76)	
B	89 (24)	
History of decompensation, *n* (%)	119 (32)	0.980
Sodium mM, median (IQR)	139 (137–141)	0.300
Creatinine mg/dL, median (IQR)	0.9 (0.8–1.2)	0.026
Creatinine quartiles, *n* (%)		0.291
≤0.8 mg/dL	136 (36)	
0.9 mg/dL	74 (20)	
1.0–1.1 mg/dL	70 (19)	
>1.2 mg/dL	93 (25)	
Bilirubin mg/dL, median (IQR)	1.0 (0.6–1.6)	0.154
Albumin g/dL, median (IQR)	3.4 (2.9–3.7)	0.002
Albumin quartiles, *n* (%)		0.006
≤2.9 g/dL	95 (25)	
3.0–≤3.4 g/dL	107 (29)	
3.5–≤3.7 g/dL	84 (23)	
>3.7 g/dL	87 (23)	
INR, median (IQR)	1.1 (1.0–1.2)	0.018
INR quartiles, *n* (%)		0.349
≤1.0	111 (30)	
1.1	116 (31)	
1.2	76 (20)	
≥1.3	70 (19)	
MELD-Na, median (IQR)	9 (7–12)	0.819
HCC Baseline		
Multifocal, *n* (%)	91 (24)	< 0.001
Index lesion cm, median (IQR)	2.8 (2.2–3.7)	< 0.001
Milan Criteria, *n* (%)	308 (83)	< 0.001
UCSF Criteria, *n* (%)	40 (11)	
T-Stage, *n* (%)		< 0.001
T1	282 (76)	
T2	91 (24)	
BCLC Stage, *n* (%)		< 0.001
A	326 (87)	
B	47 (13)	
ECOG, *n* (%)		0.118
0	228 (61)	
1	145 (39)	
AFP ng/mL, median (IQR)	13 (5.2–75)	< 0.001
AFP ≤ 20 ng/mL, *n* (%)	215 (58)	< 0.001
Index Liver-Directed Therapy		
Modality, *n* (%)		0.066
DEE-TACE	155 (42)	
90Y	136 (36)	
MWA	82 (22)	
Days from diagnosis to treatment, median (IQR)	56 (39–82)	0.556

Continuous factors reaching significance were converted to categorical quartiles for univariate confirmation. Abbreviations: interquartile range (IQR), Hepatitis C virus (HCV), nonalcoholic steatohepatitis (NASH), alcoholic liver disease (ALD), international normalized ratio (INR), University of California San Francisco (UCSF), Barcelona Clinic Liver Cancer (BCLC), Eastern Cooperative Oncology Group (ECOG), alpha fetoprotein (AFP), drug-eluting embolic transarterial chemoembolization (DEE-TACE), 90-Yittrium (90Y), microwave ablation (MWA).

**Table 2:** Multivariate analysis of time to progression by albumin quartiles.

Variate	Milan Model		T-Stage Model		BCLC Model	
Parameters	*p*-Value	HR (95% CI)	*p*-Value	HR (95% CI)	*p*-Value	HR (95% CI)
Albumin quartiles						
3.0–≤3.4 g/dL vs. ≤2.9 g/dL	0.006	2.1 (1.2–3.5)	0.004	2.2 (1.3–3.7)	0.006	2.1 (1.2–3.5)
3.5–≤3.7 g/dL vs. 3.0–≤3.4 g/dL	0.635		0.897		0.908	
>3.7 g/dL vs. 3.5–≤3.7 g/dL	0.717		0.810		0.903	
Milan Criteria						
Outside vs. Within	<0.001	2.5 (1.6–3.9)				
T-Stage						
T1 vs. T2			<0.001	2.3 (1.5–3.5)		
BCLC Stage						
A vs. B					<0.001	2.5 (1.5–4.0)
AFP						
>20 ng/mL vs. ≤…	<0.001	2.7 (1.8–4.2)	<0.001	2.8 (1.8–4.3)	<0.001	2.8 (1.8–4.3)

Abbreviations: hazard ratio (HR), 95% confidence interval (95% CI), Barcelona Clinic Liver Cancer (BCLC), alpha fetoprotein (AFP).

**Table 3:** Multivariate analysis of time to progression by albumin median split.

Variate	Milan Model		T-Stage Model		BCLC Model	
Parameters	*p*-Value	HR (95% CI)	*p*-Value	HR (95% CI)	*p*-Value	HR (95% CI)
Albumin median split						
<3.4 g/dL vs. ≥3.4 g/dL	0.001	1.9 (1.3–3.0)	0.005	1.8 (1.2–2.7)	0.005	1.8 (1.2–2.7)
Milan Criteria						
Outside vs. Within	<0.001	2.6 (1.6–4.0)				
T-Stage						
T1 vs. T2			<0.001	2.3 (1.5–3.4)		
BCLC Stage						
A vs. B					<0.001	2.6 (1.5–4.1)
AFP						
>20 ng/mL vs. ≤…	<0.001	2.7 (1.8–4.1)	<0.001	2.8 (1.8–4.3)	<0.001	2.8 (1.8–4.3)

Abbreviations: hazard ratio (HR), 95% confidence interval (95% CI), Barcelona Clinic Liver Cancer (BCLC), alpha fetoprotein (AFP).

**Table 4:** Multivariate analysis of time to progression by ALBI grade.

Variate	Milan Model		T-Stage Model		BCLC Model	
Parameters	*p*-Value	HR (95% CI)	*p*-Value	HR (95% CI)	*p*-Value	HR (95% CI)
ALBI Grade						
Grade 1 vs. Grade 2	0.144		0.225		0.127	
Grade 2 vs. Grade 3	0.037	1.9 (1.4–6.3)	0.031	1.9 (1.3–6.0)	0.061	
Milan Criteria						
Outside vs. Within	0.001	2.2 (1.4–3.3)				
T-Stage						
T1 vs. T2			0.001	2.1 (1.4–3.2)		
BCLC Stage						
A vs. B					0.002	2.3 (1.4–3.6)
AFP						
>20 ng/mL vs. ≤…	<0.001	2.8 (1.8–4.4)	<0.001	2.8 (1.9–4.4)	<0.001	2.9 (1.9–4.5)

Abbreviations: hazard ratio (HR), 95% confidence interval (95% CI), Barcelona Clinic Liver Cancer (BCLC), alpha fetoprotein (AFP).

**Table 5:** Clinical parameters associated with albumin < 3.4 g/dL.

Parameters	<3.4 g/dL	≥3.4 g/dL	*p*-Value
Cohort, *n*	176	197	
Study period, date range			
General Demographics			
Age, median (IQR)	62 (59–66)	64 (61–67)	0.003
Legal sex, n male (%)	122 (69)	164 (83)	0.002
Hepatology at Diagnosis			
Cirrhosis etiology, *n* (%)			0.735
HCV	95 (54)	113 (57)	
NASH	27 (15)	25 (13)	
HCV + ALD	20 (11)	24 (12)	
ALD	19 (11)	15 (8)	
Other	15 (9)	20 (10)	
Child Pugh, *n* (%)			<0.001
A	94 (53)	190 (96)	
B	82 (47)	7 (4)	
History of decompensation, *n* (%)	87 (49)	32 (16)	<0.001
Sodium mM, median (IQR)	138 (136–140)	140 (138–141)	<0.001
Creatinine mg/dL, median (IQR)	0.9 (0.8–1.2)	0.9 (0.8–1.1)	0.430
Bilirubin mg/dL, median (IQR)	1.4 (0.9–2.1)	0.8 (0.5–1.2)	<0.001
INR, median (IQR)	1.2 (1.1–1.3)	1.1 (1.0–1.1)	<0.001
ALBI Grade, *n* (%)			<0.001
Grade 1	0 (0)	67 (34)	
Grade 2	122 (69)	130 (66)	
Grade 3	54 (31)	0 (0)	
MELD-Na, median (IQR)	11 (8–14)	8 (7–10)	<0.001
HCC Baseline			
Multifocal, *n* (%)	44 (25)	46 (23)	0.717
Index lesion cm, median (IQR)	2.8 (2.2–3.8)	2.8 (2.2–3.7)	0.897
Milan Criteria, *n* (%)	147 (84)	161 (82)	0.683
T-Stage, *n* (%)			0.631
T1	131 (74)	151 (77)	
T2	45 (26)	46 (23)	
BCLC Stage, *n* (%)			0.876
A	153 (87)	173 (88)	
B	23 (13)	24 (12)	
ECOG, *n* (%)			0.001
0	92 (52)	136 (69)	
1	84 (48)	61 (31)	
AFP ng/mL, median (IQR)	16 (6–73)	10 (5–79)	0.142
AFP ≤ 20 ng/mL, *n* (%)	98 (56)	117 (60)	0.461
Index Liver-Directed Therapy			
Modality, *n* (%)			0.002
DEE-TACE	89 (51)	66 (34)	
90Y	50 (28)	86 (44)	
MWA	37 (21)	45 (23)	
Days from diagnosis to treatment, median (IQR)	58 (36–84)	55 (34–81)	0.398
Objective Response Rate, *n* (%)			0.131
CR/PR	108 (61)	140 (71)	
SD/DP	53 (30)	46 (23)	
NA	15 (9)	11 (6)	
Primary Endpoint	117 (66)	99 (50)	0.514
Liver transplantation, *n* (%)	63 (54)	54 (55)	
Tumor progression, *n* (%)	54 (46)	45 (45)	
Source of Tumor Progression			0.778
Index progression, *n* (%)	33 (61)	25 (56)	
New disease, *n* (%)	21 (39)	20 (44)	

Continuous factors reaching significance were converted to categorical quartiles for univariate confirmation. Abbreviations: interquartile range (IQR), Hepatitis C virus (HCV), nonalcoholic steatohepatitis (NASH), alcoholic liver disease (ALD), international normalized ratio (INR), University of California San Francisco (UCSF), Barcelona Clinic Liver Cancer (BCLC), Eastern Cooperative Oncology Group (ECOG), alpha fetoprotein (AFP), drug-eluting embolic transarterial chemoembolization (DEE-TACE), 90-Yittrium (90-Y), microwave ablation (MWA), Complete response (CR), PR (partial response), Stable disease (SD), Disease progression (DP), not available (NA).

## Data Availability

The dataset generated for the current study is available from the corresponding author on reasonable request.

## Supplementary Materials

The following supporting information can be downloaded at: www.mdpi.com/article/10.3390/cancers14071684/s1, Table S1: ALBI Grade Breakdown by Albumin Quartiles.
