# Supplementary material for: Hypoalbuminemia Is a Hepatocellular Carcinoma Independent Risk Factor for Tumor Progression in Low-Risk Bridge to Transplant Candidates
Source: Cancers (Basel). 2022 Mar 25;14(7):1684. doi: 10.3390/cancers14071684 (PMC8996921; doi:10.3390/cancers14071684)
Supplement: Supplementary file 1 [file cancers-14-01684-s001.zip › Supplement Table.pdf]

Table S1. ALBI Grade Breakdown by Albumin Quartiles.

Parameters	ALBI Grade 1	ALBI Grade 2	ALBI Grade 3
Cohort, n	67	252	54
Albumin Quartiles, n (%)			
≤ 2.9 g/dL	0 (0)	41 (16)	54 (100)
3.0 - ≤ 3.4 g/dL	0 (0)	107 (42)	0 (0)
3.5 - ≤ 3.7 g/dL	7 (10)	77 (31)	0 (0)
> 3.7 g/dL	60 (90)	27 (11)	0 (0)
